# Characterization of *IG-MYC*-breakpoints and their application for quantitative minimal disease monitoring in high-risk pediatric Burkitt-lymphoma and -leukemia

**DOI:** 10.1038/s41375-022-01626-w

**Published:** 2022-07-05

**Authors:** Paula Möker, Udo zur Stadt, Martin Zimmermann, Malik Alawi, Stephanie Mueller, Jasmin Finger, Fabian Knörr, Amambay Riquelme, Ilske Oschlies, Wolfram Klapper, Jutta Bradtke, Birgit Burkhardt, Wilhelm Woessmann, Christine Damm-Welk

**Affiliations:** 1 Pediatric Hematology and Oncology and NHL-BFM Study Center, University Medical Center Hamburg-Eppendorf, 20246 Hamburg, Germany; 2 Pediatric Hematology and Oncology and CoALL Study Center, University Medical Center Hamburg-Eppendorf, 20246 Hamburg, Germany; 3 Department of Pediatric Hematology and Oncology, Hannover Medical School, and NHL-BFM Study Center, Hannover, Germany; 4 Bioinformatics Core, University Medical Center Hamburg-Eppendorf, 20246 Hamburg, Germany; 5 Pediatric Hematology and Oncology and NHL-BFM study Center, University Hospital Muenster, Muenster, Germany; 6 Mildred Scheel Cancer Career Center HaTriCS4, University Medical Center Hamburg- Eppendorf, Hamburg, Germany; 7 Department of Pathology, Hematopathology Section and Lymph Node Registry, University Hospital Schleswig-Holstein, Campus Kiel/Christian-Albrechts-University, Kiel, Germany; 8 Department of Pathology, Justus-Liebig-University, Giessen, Germany

**Keywords:** Risk factors, B-cell lymphoma

## To the Editor:

The cure rate of children with Burkitt lymphoma (BL) and -leukemia (B-AL) reaches 90% with NHL-BFM-type chemotherapy. Only children with clinical high-risk disease (stage III or IV, LDH > 500U/l and/or CNS-involvement, risk groups R3 and R4; supplementary table 1) have a relapse rate exceeding 15% and their survival at relapse is 20% [1–4]. Therefore, early identification of children with highest risk of relapse among clinical high-risk patients is essential for further therapy optimization. The goal of our study was to analyze the prognostic value of *IG-MYC*-breakpoints from the hallmark translocations t(8;14) (*IGH-MYC*), t(2;8) (*IgK-MYC*) and t(8;22) (*IGL-MYC*) and apply them to evaluate quantitative minimal disseminated disease (MDD, for BL) or minimal residual disease (MRD, for B-AL) as risk factors among children with high-risk disease.

We determined *IG-MYC*-breakpoints in samples from children with BL/B-AL, risk groups R3 and R4, treated with identical chemotherapy in NHL-BFM studies/-registry between 2000 and 02/2017 (supplementary patient data) for whom frozen or FFPE initial tumor material, and frozen bone marrow cells from either initial punctures (93 BL, for MDD) or before the second course of chemotherapy (50 B-AL, for MRD) were available. The patient characteristics of the 143 BL/B-AL patients are shown in supplementary table 2. EFS and survival of the group were 80 ± 3% and 83 ± 3%, respectively.

The breakpoint distributions of the translocations t(8;14), t(2;8) and t(8;22) in BL/B-AL reported so far often were analyzed by methods excluding chromosome 8-breakpoints far 3´of *MYC* and/or in limited cohorts of patients due to the necessity of available frozen tumor material [5–8]. Using a long-distance PCR for *IGH-MYC* and Sanger sequencing in cases with frozen tumor samples or a genomic capture high-throughput sequencing (gc-hts) assay for *IGH-MYC*-negative cases and those with FFPE tumor DNA (supplementary methods) [9] *IG-MYC* breakpoints could be detected in 128 (90%) of the tumor samples.

**Fig. 1:**
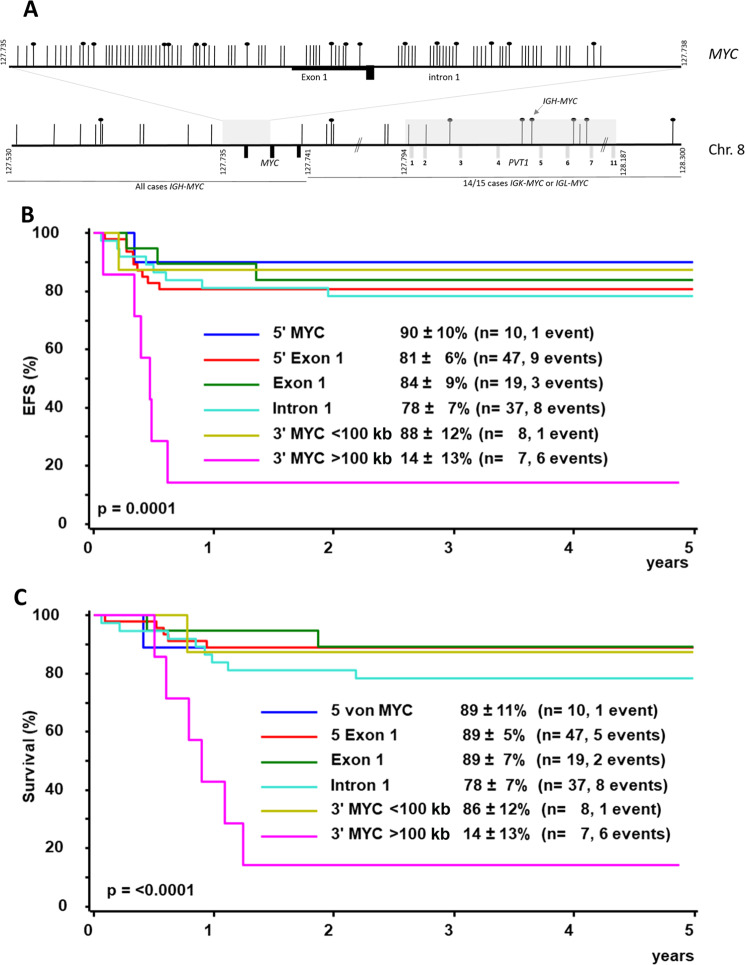
Breakpoints on chromosome 8 in 128 patients with Burkitt lymphoma or leukemia risk group R3/R4. **A** Localization of the breakpoint on chromosome 8. Each vertical line represents a patient´s breakpoint; lines with a head denote patients who relapsed. **B** Event-free survival and (**C**) survival at three years of 128 patients with Burkitt lymphoma / leukemia risk group R3/R4 according to the breakpoint on chromosome 8.

The distribution of the breakpoints on chromosome 8 among the 128 patients are consistent with earlier reports [5–8]. In total, 8% of the breakpoints were located 5ʹ of *MYC*, 37% 5ʹ of Exon 1, 15% in Exon 1, 29% in Intron 1, 6% within 100 kb 3ʹ of *MYC*, and 5% >100 kb 3ʹ of *MYC* (Fig. 1A). EFS and survival did not differ significantly between the almost 95% of patients with a breakpoint between 5´of *MYC* and 100 kb 3ʹ of *MYC*. A chromosome 8 breakpoint >100 kb 3´of *MYC* was associated with a significantly lower EFS and survival (6 of 7 patients relapsed, EFS and survival 14 ± 13%) compared to all other breakpoints (EFS 82 ± 4%, survival 86 ± 3%, *p* < 0.0001) (Fig. 1B, C).

Regarding the *IG* involved, 114 samples showed an *IGH-MYC* fusion (89%), 10 an *IGL-MYC*- (8%) and four an *IGK-MYC*- fusion (3%). As described earlier, chromosome 8 breakpoints 3´ of *MYC* were associated with *IGL-* and *IGK-MYC*-fusions (14/14 samples compared to 1/114 samples with *IGH-MYC*, *p* < 0.0001) [6–8]. *IGL*-fusions correlated with a breakpoint far 3´of *MYC* ( > 100 kb), as well (5/10 samples, *p* < 0.0001) (Fig. 1A). Children with an *IGL-MYC* fusion had a significantly lower EFS and survival compared to those with *IGH-MYC* fusion (EFS, 50 ± 26% compared to 81 ± 4%, *p* = 0.013; survival, 50 ± 26% compared to 85 ± 4%, *p* = 0.017; supplementary Fig. 1). Among the four children with *IGK-MYC* fusion, one child relapsed and died.

Given that less than 1% of *IGH-MYC* breaks were 3ʹ of *MYC*, rapid and cost-effective detection of the small very high-risk group of patients with a chromosome 8 breakpoint >100 kb 3ʹ of *MYC* within the plasmocytoma variant translocation 1 (*PVT1*) gene can be facilitated by a pre-screen using fluorescence in-situ hybridization for an *IGH*-break.

The Italian Association of Pediatric Hematology and Oncology (AIEOP) detected MDD in 30% of *IGH-MYC*-positive children with high-risk BL and initial frozen tumor using the semi-quantitative long-distance PCR with a detection limit of 10^−4^ to 10^−3^ for minimal disease evaluation. The progression-free survival of MDD-positive high-risk patients was 68% compared to 93% for all other patients in risk-group R4 [10, 11]. Persistence of MRD after the first course of chemotherapy in 20% of patients with B-AL was associated with a significantly reduced progression-free survival, as well [12, 13]. Limitations of the long-distance PCR for *IGH-MYC* are the applicability to only 2/3 of children with BL/B-AL, the necessity for frozen initial tumor material and its semi-quantitative nature.

A minimal disease marker could be established for all children with BL/B-AL in our study by using the *IG-MYC* fusions (128 patients) and clonal *IG*-rearrangements for the remaining 15 patients. Our approach allowed overcoming the limitations of *IGH-MYC* long-distance PCR, by extending minimal disease analysis to patients without *IGH-MYC*-fusion and those without available frozen tumor material [10–13]. According to the patient-specific genetic or clonal rearrangement, quantitative dPCR assays with a quantitative range of 10^−4^ were established for minimal disease detection (supplementary methods, supplementary Fig. 2 and supplementary results).

**Fig. 2:**
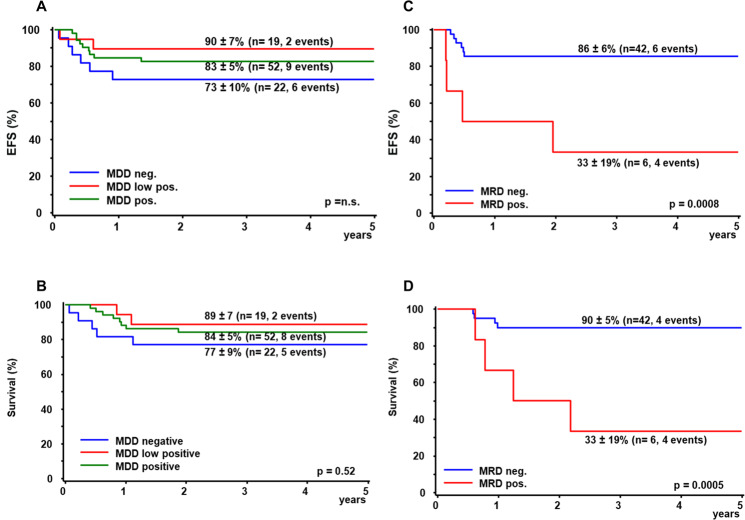
Outcome of Burkitt Lymphoma and Burkitt Leukemia patients according to quantitative minimal disease measurement in bone marrow. Event-free survival (**A**, **C**) and survival (**B**, **D**) at three years of 93 patients with Burkitt lymphoma risk group R3/R4 according to minimal disseminated disease (MDD, **A**, **B**) and of 48 patients with Burkitt leukemia Risk group R3/R4 according to minimal residual disease (MRD, **C**, **D**).

An *IG-MYC*-fusion was used as MDD-marker in 79 patients with BL, and clonal *IG*-rearrangements in 14 patients. MDD was positive in 72 of 93 initial bone marrow samples of BL-patients risk group R3 and R4 (76%). In 21 samples, MDD was low positive, in 51 MDD ranged from 1.2 × 10^−4^ to 2.8 × 10^−1^. The quantity of cytological bone marrow infiltration correlated with quantitative MDD (Spearman´s rho 0.79, *p* < 0.001, supplementary Fig. 3). The higher percentage of MDD-positivity in our study compared to 30% among all BL-patients and 40% among R4-patients in the AIEOP-studies can be attributed to the higher sensitivity of our dPCR-assay [10, 11]. In contrast to the results of AIEOP, neither detection of MDD nor positivity within the quantitative range were associated with EFS or survival among high-risk BL-patients in our study (Fig. 2A, B). This was also the case for patients in risk group R4 (supplementary Fig. 4). This difference to the AIEOP study results cannot be readily explained by the higher detection rate of MDD in our study: The relapse risk did not correlate with MDD even among R4-patients with MDD in the quantitative range (55% of R4-patients), and the relapse risk did not rise with increasing MDD. Both studies analyzed a large cohort of identically treated patients with comparable outcome so that a selection bias besides restriction to *IGH-MYC* positive BL in the AIEOP-study is unlikely to account for the observed difference. Taken together, we cannot explain the diverging results on the prognostic meaning of MDD in children with BL. Further studies on MDD in BL are needed to unravel its role as a prognostic marker.

MRD before the second course of BFM-type chemotherapy could be analyzed in 48 of 50 children with B-AL (47 by *IG-MYC*-fusion, 1 by clonal *IG*-rearrangement). Two patients with negative dPCR-result were excluded due to low control gene amplification. EFS and survival of the six patients with detectable MRD was 33 ± 19% compared to 86 ± 6% and 90 ± 5%, respectively, for the 42 patients without MRD (*p* < 0.001) (Fig. 2C+D). MRD was low-positive in four samples and 8 × 10^−2^ and 15 × 10^−2^ in the remaining two patients, of which only the latter showed cytological persistence of L3-blasts. Our results are in line with those reported using the long-distance PCR for *IGH-MYC* by the AIEOP [12, 13]. Despite the different methodologies the sole detection of early MRD and not its quantity was associated with a very high risk of relapse of more than 60% among children with B-AL [13].

Whether this finding still holds true for treatment regimen including rituximab cannot be answered by our MRD-analysis in additional nine children with B-AL who received rituximab chemo-immunotherapy since none of the children relapsed (follow-up 6–88 months, median 65) with low-positive MRD detectable in one child. MRD-data from the AIEOP on 21 patients (3 positive) who received rituximab-containing chemo-immunotherapy may hint towards this possibility [12]. However, generalization of the data is hampered by a progression-free survival of less than 80% compared to more than 90% reported with chemo-immunotherapy in larger series [12, 14].

Although we were able to analyze a large group of high-risk BL/B-AL patients all treated identically for MDD and MRD, patient selection by material-availability as well as sample collection over a long time period are limitations of our study.

Distinguishing relapse from second malignancy is of utmost importance in children with BL/B-AL since second malignancies may be treated successfully by front-line therapy while patients with BL/B-AL-relapse currently have a very poor outcome [1, 4, 15]. Despite clonality analysis, differentiation remains difficult in some cases. The tumor- and patient-specific genomic *IG-MYC* breakpoint enabled distinguishing relapse from second malignancy in three children (supplementary Fig. 5).

In summary, our results show that chromosome 8 breakpoints far distal of *MYC* correlate with poor survival in children with high-risk BL/B-AL. This finding needs corroboration in larger cohorts, and biological studies are necessary to uncover its molecular basis. Due to its close association with *IGL*- and *IGK*-fusions, screening could be performed by fluorescence in-situ hybridization for t(8;14) followed by sequencing of *MYC* in *IGH*-negative cases. A minimal disease marker can be established for almost all children with BL/B-AL by combining LD-PCR for *IGH-MYC* and a customized gc-hts-assay. Using a quantitative dPCR-assay the prognostic value of MRD could be confirmed for children with B-AL, whereas further studies are necessary to evaluate the meaning of MDD in children with BL.

## Supplementary information

Supplementary data

Supplementary Table 3
