# Supplementary material for: Characterization of IG-MYC-breakpoints and their application for quantitative minimal disease monitoring in high-risk pediatric Burkitt-lymphoma and -leukemia
Source: Leukemia. 2022 Jul 5;36(9):2343–6. doi: 10.1038/s41375-022-01626-w (PMC9417994; doi:10.1038/s41375-022-01626-w)
Supplement: Supplementary file 1 — Supplementary data [file 41375_2022_1626_MOESM1_ESM.docx]

**Supplementary Patients and Methods**

*Patients*

Patients diagnosed with confirmed BL/B-AL from Germany included in ethically approved studies or registries of the NHL-BFM study group between 01/2000 and 02/2017 (NHL-BFM 95 (N95), B-NHL BFM 04 (B04) or the NHL-BFM Registry 2012 (REG12) were stratified according to clinical stage, initial resection status, LDH level and CNS-involvement into four risk groups (R1 – R4) (definition: supplementary table 1). Patients in risk groups R3 and R4 (BL/B-AL stage III or IV, LDH >500U/l and/or CNS-involvement) not treated by rituximab were potentially eligible for this study. The treatment intensity and the drugs were comparable among the study protocols^1, 2^. From study NHL-BFM 95, only patients who received 5g/m^2^ MTX given for 24 h (standard arm) were included^1^.

Central reference histology or cytology included immune histology or flow-cytometry. In 14 of 15 cases with no detectable *IG-MYC*-fusion by our molecular diagnostics a *MYC*-break was confirmed by fluorescence in-situ hybridization.

Eligibility further included the availability of a) frozen or FFPE initial tumor material, b) for children with BL initial frozen bone marrow mononuclear cells for MDD and c) for those with B-AL frozen bone marrow from before the second course of chemotherapy for MRD.

Staging bone marrow smears and CSF-cytospins were centrally reviewed. Initial staging and adequate risk-adapted therapy were monitored by the NHL-BFM study center. All patients or their parents gave informed consent for transfer of their data to the NHL-BFM study center.

Altogether, 93 patients with BL and 50 patients with B-AL met the described criteria and were included in the study (n=143).

In addition to these study patients, MRD could be analyzed in 9 patients with B-AL who received rituximab outside of a study before the first course of NHL-BFM chemotherapy.

*Workflow*

Initial frozen tumor samples were analyzed by LD-PCR to detect *IGH-MYC*-rearrangements. In samples positive for *IGH-MYC* the genomic breakpoint was sequenced by Sanger sequencing. LD-PCR negative samples and all FFPE tumor samples were analyzed by gc-hts. Following the manufacturer's instructions, high-molecular-weight genomic DNA (gDNA) was extracted from nucleated cells, frozen tumor cells and FFPE samples using DNA Blood Mini-, QIAamp DNA- or AllPrep DNA/RNA FFPE- Kit (Qiagen, Hilden, Germany) respectively.

According to the patient-specific genetic rearrangement, quantitative assays for dPCR were established for minimal disease detection.

Adequate frozen tumor samples were available for 50 B-AL and 71 BL patients. In addition, FFPE samples were used in 13 BL patients.

*Marker screening by LD-PCR for IGH-MYC and Sanger sequencing*

All initial samples eligible for LD-PCR were analyzed for *IGH-MYC* using the GoTaq® Long PCR Master Mix (Promega, Walldorf, Germany). A multiplex PCR reaction with one primer for *MYC* and four primers for each breakpoint region in the constant regions *Cµ*, *Cγ*, *Cα*, and the *IgH* enhancer region, was performed (adapted from ^3-7^, supplementary table 3A).The PCR reaction was carried out with 150 ng of gDNA, 0.2µM of each primer and 2x GoTaq® Long PCR Master Mix according to the manufacturer´s instructions. Initial denaturation was followed by 30 cycles with denaturation for 30 seconds at 94°C and annealing/extension for 8 minutes at 65°C and a final extension for 5 minutes at 72°C. The genomic quality and feasibility of long-distance amplification were analyzed with control primers for a 17.5kb β-Globin fragment (Promega, Walldorf, Germany). PCR products were analyzed on agarose gels. Samples with a positive LD-PCR result for *IGH-MYC* -rearrangement underwent Sanger sequencing using the BigDye™ Direct Cycle Sequencing Kit (ThermoFisher Scientific, Darmstadt, Germany) (sequencing primers, supplementary table 3B). Patient-specific *IGH-MYC*-breakpoints were identified using BLAST homology search^8^.

*Marker screening by gc-hts for IG-MYC- and clonal Ig-rearrangements*

A custom gc-hts assay to detect either *MYC*-associated genomic breakpoints or IG-related clonal rearrangements, was based on a previously published and extended panel that finally encompassed 955Kb of genomic sequences^9^**.** To examine the entire *MYC* locus on chromosome 8, additional 80-mer capture probes were selected comprising 11Kb of genomic sequences that include a 5Kb region immediately upstream of *MYC* exon 1 (supplementary table 3C). For detection of breakpoints far 5‘ or 3‘ of *MYC* and for the detection of clonal rearrangements*,* capture probes specific for different (diversity-), joining- and constant-regions on chromosome 2 (*IGK*), chromosome 22 (*IGL*) and chromosome 14 (*IGH*_DH-M) were selected. Final probe design was performed by Illumina Concierge Service (Illumina, San Diego, CA).

For library preparation the Nextera DNA flex protocol (Illumina) with 400ng of gDNA or 200ng of FFPE DNA was used according to the manufacturer guidelines and recommendations. Twelve pre-enriched libraries (8 for FFPE DNA) were pooled by volume for the target-specific enrichment. A Nextseq 500/550 with mid output 300 cycle v2.5 kits were used for paired-end sequencing.

Detection of translocation breakpoints and fusion sequences with the open-source software segemehl have been described previously^10, 11^. Clonal rearrangements were identified using the open-source platform Vidjil ([www.vidjil.org](http://www.vidjil.org))^12^. All genomic coordinates provided in this study refer to version hg38 of the human reference assembly.

*Detection of MDD and MRD by digital droplet PCR*

Patient-specific dPCR assays were selected from the genomic *IG-MYC* breakpoints or *IG* related clonal rearrangements. MDD and MRD in bone marrow samples of BL/B-AL patients was performed and analyzed by dPCR according to Drandi et al. using the QX200 digital PCR system (BIO-RAD, Munich)^13^.

For each MDD/MRD analysis, 500 ng gDNA from patients' BM-MNCs and negative control DNA (pooled gDNA from 5-10 pooled PB-MNCs from healthy individuals) were analyzed in triplicates. No template controls were analyzed in duplicates. A positive control (10^-1^ dilution of the initial tumor samples) was analyzed in an additional well. In parallel, DNA quality was controlled through amplification of the *β-globin* house-keeping gene in one additional well. Each reaction contained 10µl 2X ddPCR™ supermix for probes (no dUTP, BIO-RAD, Munich, Germany), 500ng of genomic DNA, 500nM of each primer (Eurofins Genomics, Ebersberg, Germany) 200nM of probe (double-quenched Probes with 5' 6-FAM™ as reporter dye and Zen™ and 3' Iowa-Black®FQ as a quencher, Integrated DNA Technologies, Leuven, Belgium, or 5' 6-FAM™as reporter and 3'BHQ1 quenched Metabion, Planegg, Germany; supplementary table 2b) and 2 Units restriction enzyme *HindIII* or *EcoRI* (NEW ENGLAND BioLabs, Frankfurt, Germany). Probes and primers are listed in supplementary table 3D. Optimization of dPCR conditions using temperature gradients from 61.2 to 55.8°C were performed for all allele-specific Primers, using the 500 ng of the 10^-1^ to 10^-2^ dilutions of the initial diagnostic sample per well. Sensitivity assessment of the patient-specific assays was performed with 500 ng of the 10^-4^ dilution of the diagnostic DNA samples. The dPCR was conducted under the following conditions: 95°C,10 minutes for enzyme, 44 cycles with denaturation at 94°C for 30 seconds, and annealing and extensions with assay-specific temperature for 1 minute, and a final signal stabilization at 98°C for 10 minutes. For longer PCR products ≥250 bp, a three-step cycling protocol was applied, adding an additional extension step at 72°C for 2 minutes, after the assay-specific annealing extension step in the two-step protocol.

*Data analysis of dPCR*

Data for dPCR were analyzed with QuantaSoft™ software Version 1.7 (BIO-RAD, Munich) according to Drandi et al.^13^. Only replicates with ≥9000 droplets were eligible for data analysis. The threshold was placed below the positive control droplet group and close to the negative background and suspect false positive droplets were excluded in the second fluorescence channel of the QuantaSoft™ software. A merge of three or more positive droplets in all three replicates was considered MDD/MRD positive. A merge of two droplets in triplicate is considered positive below the quantitative limit of the assay (BQL). Sample with no or one positive droplet is considered as MDD/MRD negative. DNA samples with >300 and <7500 copies/µl in the β-globin housekeeping gene were considered as evaluable for the study.

Positive dPCR results were calculated using the mean value of the copy-number/µl of the replicates multiplicated with the reaction volume of 20 µl. If the negative control had more than three positive droplets and no alternative allele-specific primer could be established, then the mean copy number/20 µl of the negative control sample was subtracted from the mean copy number of the MDD/MRD sample.

For statistical analysis, the dPCR results are expressed as target copies per 10^5^ cells, assuming that 500 ng of genomic DNA corresponds to 75000 cells.

The quantitative range was determined for each patient’s specific dPCR assay. Positive dPCR results below the quantitative range (BQR) or limit (BQL) of the individual assay were defined as low positive.

The quantitative range of the individual dPCR assays reached 1 x10^-3^ for one patient, 5 x10^-4^ for 12 patients and 1 x10^-4^ for 130 patients. The sensitivity of minimal disease detection for dPCR was 100fold higher compared to LD-PCR in two patients analyzed by both methods (supplementary Fig. 2 and supplementary results).

*Statistical analysis*

Univariate analysis was conducted by the Wilcoxon test for quantitative variables and Fisher's exact test for qualitative variables. When frequencies were sufficiently large, χ^2^-test was used. Event-free survival (EFS) was calculated from date of diagnosis to last follow-up or first event (relapse, secondary malignancy or death of any cause). Survival was calculated from date of diagnosis to death of any cause or last follow-up. Probabilities of survival were estimated using the Kaplan-Meier method with standard errors according to Greenwood and were compared with the log-rank test.

Computations were performed using SAS (Statistical Analysis System Version 9.4, SAS Institute Inc, Cary, NC).

**Supplementary Results**

*Comparison of LD-PCR with dPCR for minimal disease detection in two patients.*

For two patients the sensitivity of MDD measurement was compared to LD-PCR (supplementary figure 2). The quantitative range of the two patient-specific IGH-MYC assays were 10^-1^ and 10^-2^, respectively (supplementary figure 2A), with LD-PCR and 1x 10^-4^ with dPCR for both patients. MDD was LD-PCR negative in both cases (supplementary figure 1A and C), whereas one patient was positive by dPCR (1.5 x10^-3^) and one patient was low positive (1,5x10^-5^) (supplementary figure 2B and C).

**Supplementary Table 1.** Definition of Risk groups in the NHL-BFM studies for mature B-NHL

| risk group | resection status | stage and initial serum LDH level |
| --- | --- | --- |
| R1 | complete |  |
| R2 | incomplete | I +II  stage III and LDH < 500U/L |
| R3 | incomplete | stage III and LDH ≥500 U/L but< 1000 U/L  stage IV B-AL and LDH <1000 U/L and CNS negative |
| R4 | incomplete | stage III and LDH ≥ 1000 U/L  stage IV B-AL and LDH ≥ 1000 U/L and CNS negative |
| R4 CNS+ |  | CNS positive |

LDH, lactate dehydrogenase, U/L, units/liter, CNS, central nervous system,

**Supplementary Table 2** Characteristics of 143 patients with Burkitt Lymphoma / Leukemia risk group R3/R4 analyzed for *IG-MYC* rearrangement, minimal disseminated (MDD) or minimal residual disease (MRD).

|  |  | all | BL | B-AL |
| --- | --- | --- | --- | --- |
| All patients |  | 143 | 93 | 50 |
| gender | male | 119 | 78 | 41 |
|  | female | 24 | 15 | 9 |
| Median age at diagnosis | Years  (Q1-Q3) | 9.5  (6.1-12.5) | 9.9  (6.6-12.8) | 8.7  (6.1-11.2) |
| Initial stage* | III | 69 | 69 | - |
|  | IV | 23 | 23 | - |
|  | B-AL | 50 | - | 50 |
|  | n.a. | 1 | 1 |  |
| BM | Neg | 76 | 75 | - |
|  | Pos | 64 | 15 | 49 |
|  | n.a. | 3 | 3 |  |
| CNS | Neg | 111 | 82 | 29 |
|  | Pos | 32 | 11 | 21 |
| LDH | <500 | 9 | 7 | 2 |
|  | ≥500 | 134 | 86 | 48 |
| Study / Registry | N95 | 15 | 10 | 5 |
|  | B04 | 72 | 48 | 24 |
|  | REG12 | 56 | 35 | 21 |
| Initial therapy branch | R3 | 48 | 40 | 8 |
|  | R4 | 84 | 48 | 36 |
|  | R4, CNS pos | 11 | 5 | 6 |

BM, bone marrow, CNS, central nervous system, BL, Burkitt lymphoma, B-AL, Burkitt leukemia, n.a., not available, LDH, lactate dehydrogenase, N95, study NHL-BFM 95, B04, study B-NHL BFM 04, REG12, NHL-BFM Registry 2012

*according to St. Judes´s staging system.

**Supplementary figures**


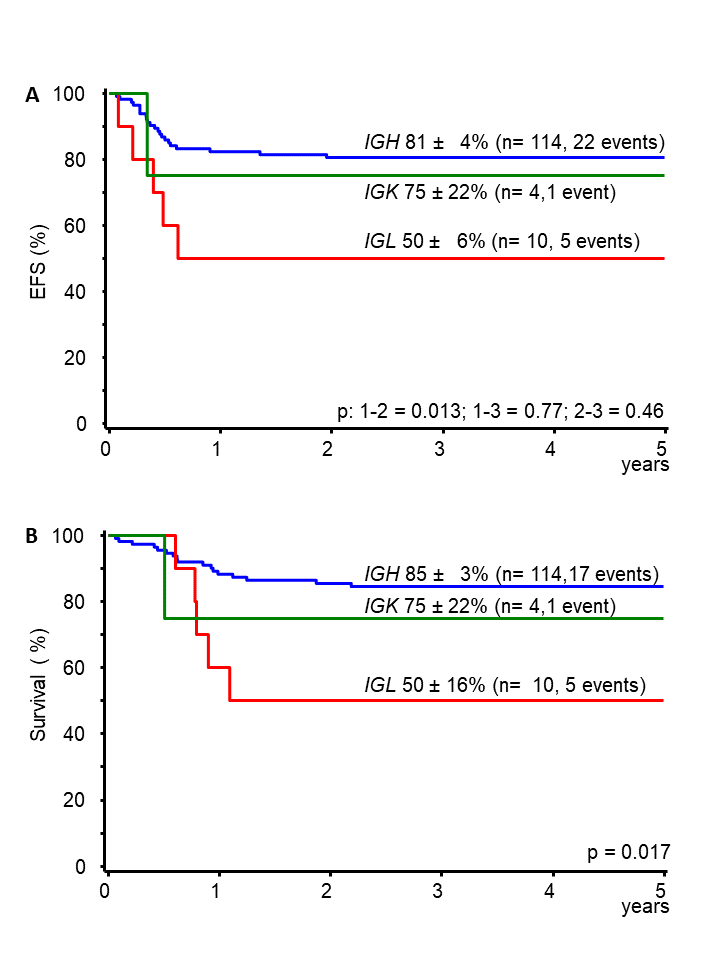


**Supplementary Figure 1.** **A** Event-free survival (EFS) and **B** survival at three years of 128 patients with Burkitt lymphoma / leukemia Risk group R3/R4 according to the *immunoglobuline*-partner of *MYC*.


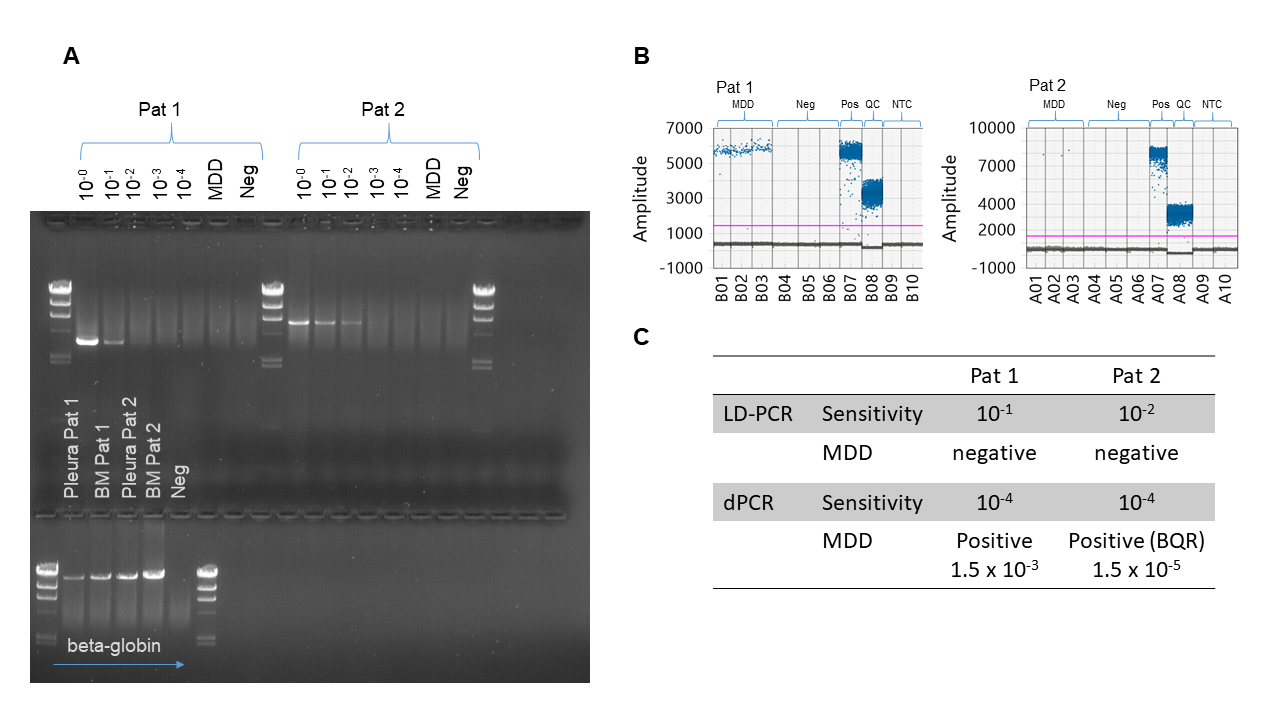


**Supplementary Figure 2.** Comparison of LD-PCR and dPCR for minimal disease quantification in two patients. **A** Gel-image of LD-PCR results of two patients. Initial pleura effusion diluted from 10^-1^ to 10^-4^ to estimate sensitivity. A 17.5 kb beta-globin amplicon was used for quality control and was detected in all analyzed samples. **B** One color plot dot outputs of MDD dPCR analysis of two patients. **C** Comparison of qualitative and quantitative PCR results. (MDD, minimal disseminated disease, Pat, patient, BM, bone marrow, LD-PCR; long distance PCR, dPCR, digital PCR, neg, negative control, pos, positive control, QC, quality control of DNA with beta-globin dPCR assay, NTC, no template control, BQR, below quantitative range.)


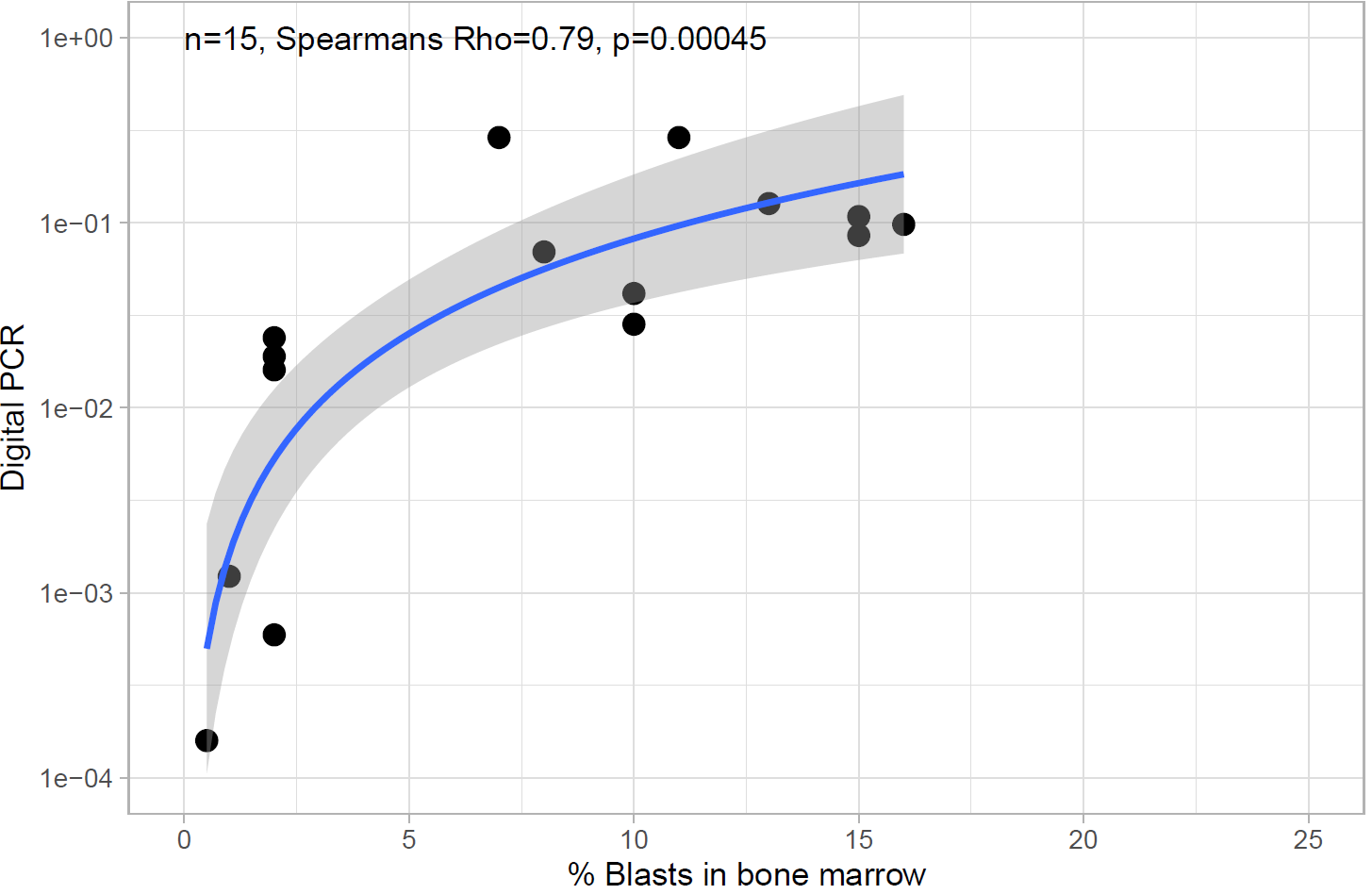


**Supplementary Figure 3.** Comparison of cytologic bone marrow involvement and quantitative digital PCR results in 15 patients with Burkitt lymphoma stage IV with bone marrow involvement. The dPCR results are expressed as the number of target molecules per 75000 cells and are shown on a logarithmic scale. Bone marrow cytological involvement is expressed as the percentage of blasts found in bone marrow smears and is shown on a linear scale.

A logarithmic compensation scale is represented by the blue line. n, number of patients.


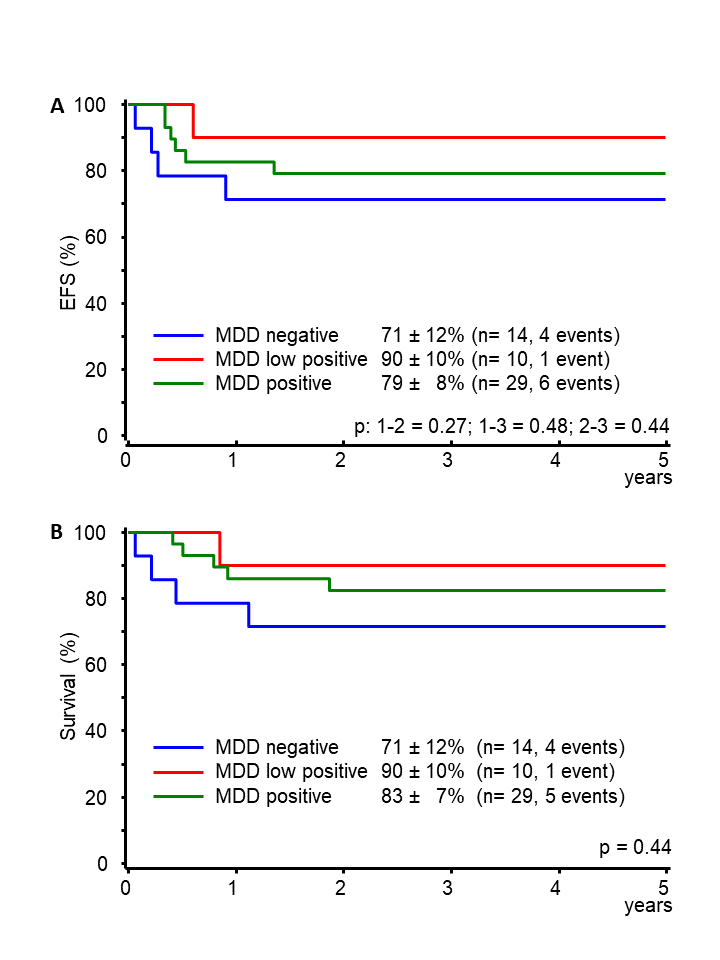


**Supplementary Figure 4.** Event-free survival (**A**) and survival (**B**) at three years of 53 patients with Burkitt lymphoma Risk group R4 according to minimal disseminated disease (MDD). (EFS, event free survival, low positive= positive not quantifiable)

**
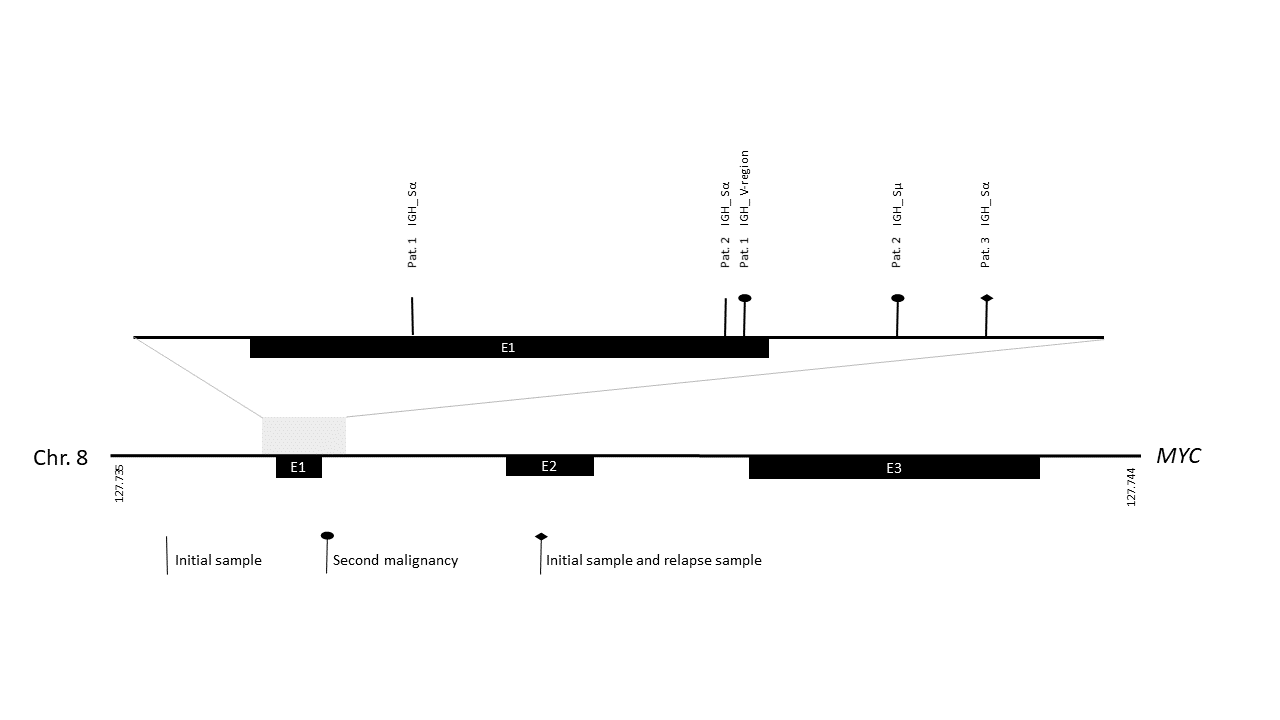
**

**Supplementary Figure 5** *IG-MYC* breakpoints from the initial and “relapse/second malignancy” samples in two patients with second malignancies and one patient with relapse.

**Supplementary References:**

1. Woessmann W, Seidemann K, Mann G, Zimmermann M, Burkhardt B, Oschlies I*, et al.* The impact of the methotrexate administration schedule and dose in the treatment of children and adolescents with B-cell neoplasms: a report of the BFM Group Study NHL-BFM95. *Blood* 2005 Feb 1; **105**(3)**:** 948-958.

2. Reiter A, Schrappe M, Tiemann M, Ludwig WD, Yakisan E, Zimmermann M*, et al.* Improved treatment results in childhood B-cell neoplasms with tailored intensification of therapy: A report of the Berlin-Frankfurt-Munster Group Trial NHL-BFM 90. *Blood* 1999 Nov 15; **94**(10)**:** 3294-3306.

3. Mussolin L, Basso K, Pillon M, D'Amore ES, Lombardi A, Luzzatto L*, et al.* Prospective analysis of minimal bone marrow infiltration in pediatric Burkitt's lymphomas by long-distance polymerase chain reaction for t(8;14)(q24;q32). *Leukemia* 2003 Mar; **17**(3)**:** 585-589.

4. Busch K, Borkhardt A, Wossmann W, Reiter A, Harbott J. Combined polymerase chain reaction methods to detect c-myc/IgH rearrangement in childhood Burkitt's lymphoma for minimal residual disease analysis. *Haematologica* 2004 Jul; **89**(7)**:** 818-825.

5. Busch K, Keller T, Fuchs U, Yeh RF, Harbott J, Klose I*, et al.* Identification of two distinct MYC breakpoint clusters and their association with various IGH breakpoint regions in the t(8;14) translocations in sporadic Burkitt-lymphoma. *Leukemia* 2007 Aug; **21**(8)**:** 1739-1751.

6. Mussolin L, Pillon M, Conter V, Piglione M, Lo Nigro L, Pierani P*, et al.* Prognostic role of minimal residual disease in mature B-cell acute lymphoblastic leukemia of childhood. *J Clin Oncol* 2007 Nov 20; **25**(33)**:** 5254-5261.

7. Mussolin L, Pillon M, d'Amore ES, Conter V, Piglione M, Lo Nigro L*, et al.* Minimal disseminated disease in high-risk Burkitt's lymphoma identifies patients with different prognosis. *J Clin Oncol* 2011 May 1; **29**(13)**:** 1779-1784.

8. Altschul SF, Gish W, Miller W, Myers EW, Lipman DJ. Basic local alignment search tool. *J Mol Biol* 1990 Oct 5; **215**(3)**:** 403-410.

9. Zur Stadt U, Alawi M, Adao M, Indenbirken D, Escherich G, Horstmann MA. Characterization of novel, recurrent genomic rearrangements as sensitive MRD targets in childhood B-cell precursor ALL. *Blood Cancer J* 2019 Nov 29; **9**(12)**:** 96.

10. Hoffmann S, Otto C, Doose G, Tanzer A, Langenberger D, Christ S*, et al.* A multi-split mapping algorithm for circular RNA, splicing, trans-splicing and fusion detection. *Genome Biol* 2014 Feb 10; **15**(2)**:** R34.

11. Stadt UZ, Escherich G, Indenbirken D, Alawi M, Adao M, Horstmann MA. Rapid Capture Next-Generation Sequencing in Clinical Diagnostics of Kinase Pathway Aberrations in B-Cell Precursor ALL. *Pediatr Blood Cancer* 2016 Jul; **63**(7)**:** 1283-1286.

12. Duez M, Giraud M, Herbert R, Rocher T, Salson M, Thonier F. Vidjil: A Web Platform for Analysis of High-Throughput Repertoire Sequencing. *PLoS One* 2016; **11**(11)**:** e0166126.

13. Drandi D, Alcantara M, Benmaad I, Sohlbrandt A, Lhermitte L, Zaccaria G*, et al.* Droplet Digital PCR Quantification of Mantle Cell Lymphoma Follow-up Samples From Four Prospective Trials of the European MCL Network. *Hemasphere* 2020 Apr; **4**(2)**:** e347.
